# ECAT11/L1td1 Is Enriched in ESCs and Rapidly Activated During iPSCGeneration, but It Is Dispensable for the Maintenance and Induction of Pluripotency

**DOI:** 10.1371/journal.pone.0020461

**Published:** 2011-05-26

**Authors:** Kumiko A. Iwabuchi, Tatsuya Yamakawa, Yoshiko Sato, Tomoko Ichisaka, Kazutoshi Takahashi, Keisuke Okita, Shinya Yamanaka

**Affiliations:** 1 Center for iPS Cell Research and Application (CiRA), Kyoto University, Kyoto, Japan; 2 Department of Stem Cell Biology, Institute for Frontier Medical Sciences, Kyoto University, Kyoto, Japan; 3 Yamanaka iPS Cell Special Project, Japan Science and Technology Agency, Kawaguchi, Japan; 4 Gladstone Institute of Cardiovascular Disease, San Francisco, California, United States of America; University of Bristol, United Kingdom

## Abstract

The principal factors that lead to proliferation and pluripotency in embryonic stem cells (ESCs) have been vigorously investigated. However, the global network of factors and their full signaling cascade is still unclear. In this study, we found that ECAT11 (L1td1) is one of the ESC-associated transcripts harboring a truncated fragment of ORF-1, a component of theL1 retrotransposable element. We generated an *ECAT11* knock-in mouse by replacing its coding region with green fluorescent protein. In the early stage of development, the fluorescence was observed at the inner cell mass of blastocysts and epiblasts. Despite this specific expression, ECAT11-null mice grow normally and are fertile. In addition, ECAT11 was dispensable for both the proliferation and pluripotency of ESCs.We found rapid and robust activation of *ECAT11* in fibroblasts after the forced expression of transcription factors that can give rise pluripotency in somatic cells.However, iPS cells could be established from ECAT11-null fibroblasts. Our data demonstrate thedispensability of ECAT11/L1td1 in pluripotency, despite its specific expression.

## Introduction

Embryonic stem cells (ESCs) have been established from mammalian blastocysts [1],[2],[3]. ESCs have the ability to proliferate vigorously and differentiate into various cell types. Therefore, they are attractive sources for cell transplantation therapy and basic research. ESCs have been used for functional analyses of numerous genes and differentiation processes. Recently, induced pluripotent stem cells (iPSCs) were derived from mouse and human somatic cells that have similar differentiation potential to ESCs, and can overcome the ethical problems and immune rejection associated with ESCs [4],[5],[6].

The molecular mechanisms and pathways underlying the pluripotency and proliferation of ESCs and iPSCs are still unclear. In mouse ESCs, pluripotency can be maintained by leukemia inhibitory factor (LIF) and several transcription factors. LIF activates Stat3 signaling and its downstream cascades [7] that are involved in pluripotency. Oct4 [8], Sox2 [9] and Nanog [10],[11] are also pivotal regulators, and maintain the undifferentiated state of ESCs. Klf4 [12] is also an important factor for the maintenance of ESCs. The Kluppel-like factor (Klf) family, involving Klf4, Klf2 and Klf5, regulates the self-renewal of ESCs [12]. Therefore, pluripotency is maintained by the regulatory networks of many transcription and other factors.

To identify new genes involved in the molecular network of pluripotency, we have previously performed a digital differential display analysis (DDD) of the expressed sequence tag libraries among various mouse tissues and cell lines [10],[13],[14],[15],[16],[17]. Candidates were selected based on their specific expression in ESCs, and included many well-known pluripotency related genes, such as Oct4 and Nanog, as well as a variety of novel genes which we designated the “ECATs” for ES cell-associated transcripts. We have shown that ECAT4 encodes the transcription factor Nanog, which plays critical roles in pluripotency [10], whereas ECAT5 encodes Eras, which promotes the proliferation of mouse ESCs [14].

In this study, we evaluated the expression and function of another ECAT, ECAT11, also known as L1ltd1. Wegenerated ECAT11 knock-outmice and ES cells by inserting the enhanced green fluorescent gene (EGFP) cDNA into the ECAT11 locus. Our study showed that ECAT11 is dispensable for the development and maintenance of pluriptotency, despite its specific expression pattern. We also found that ECAT11 is rapidly activated by Oct3/4, Sox2 and Klf4 in fibroblasts, but is dispensable for the generation of iPSCs.

## Results

### ECAT11 expression, protein structure and localization in mouse ESCs

**Figure 1:**
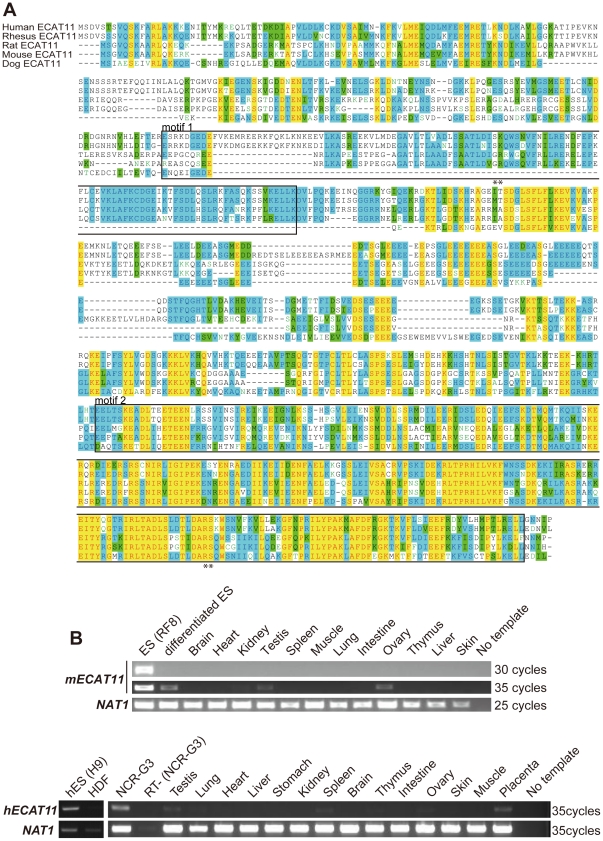
Protein structure andexpression of ECAT11. (A) Amino acid sequences of ECAT11 from various species. The boxes indicate the conserved Transposase_22 motif. Double asterisks indicate equivalent regions of arginin residues responsible for the RNA binding activity of L1ORF1. Black letters on a white background; non-similar residues, blue letters on a cyan background; consensus residues derived from a block of similar residues at a given position, black letters on a green background; consensus residues derived from the occurrence of >50% of a single residue at a given position, red letters on a yellow background; consensus residues derived from a completely conserved residue at a given position, green letters on a white background; residues weakly similar to the consensus residue at a given position. (B)The expression profiles of mouse (upper)and human (lower) ECAT11. RNA was isolated from ESCs, differentiated ESCs, human ESCs (H9), human dermal fibroblasts (HDF), an embryonic tumor cell line (NCR-G3)and various tissues from adult mice or humans, and were used for an RT-PCR analysis. The differentiation of mouse ESCs was achieved by retinoic acid (RA) treatment (300 nM) for 7 days. The amplification cycles are shown at right. NAT1 was used as a loading control.

We identified ECAT11 by a digital differential display analysis of expressed sequence tag (EST) databases [10] as a novel transcript enriched in mouse ESCs. The predicted ECAT11 open reading frame encodes 848 amino acids, showing a similarity to ORF-1, one of the two protein components of the L1 transposable element (L1). L1 is a non-LTR type of retrotransposon, which can move within the genome as a transcribed RNA intermediate. There are >599,000 copies of L1 that occupy 19% of the mouse genome [18]. L1 encodes two proteins, ORF1 and ORF2. ORF1 has a Transposase_22 motif, which is responsible for the RNA binding activity of ORF1 [19],[20],[21]. ORF2 encodes a protein with an endonuclease and reverse-transcriptase activity. ORF1 is mainly localized inthe cytoplasm as an RNA-protein particle, together with ORF2, and supportsthe L1 transition [22]. In the C-terminus of ECAT11, we found high homology with the C-terminus ofthe ORF of L1 T_Fspa_, a subtype of L1 [23]. The identity of the protein sequence in this region was 33% (Figure 1A motif2). The N-terminus of ECAT11 also showed homology with Transposase_22, withan identity of 30% (Figure 1A motif1).

ECAT11 paralogs can be identified in humans, rhesus macaques, rats and dogs (Figure 1A). The overall identity of these proteins to mouse ECAT11 is relatively low (∼23.1%), but the second ORF1-like domain showed 45.8% identity with the mouse protein. The identity of the first ORF1-like domain is 43.4%, excluding dog ECAT11, which lacks this region. However, two arginine residues (R297 and R298) of the L1 T_Fspa_ ORF1, which are critical for its RNA binding and chaperone activity [24], are not conserved in ECAT11. Therefore, it was unclear whether ECAT11 hadRNA binding and chaperone activity.

An RT-PCR analysis confirmed that ECAT11 is abundantly expressed in mouse ESCs and is suppressed upon differentiation induced by retinoic acid (Figure 1B). The expression was undetectable in most of adult somatic tissues, however, weak expression was observed in the testes, ovaries, and brain. In humans, ECAT11 transcripts were identified in ESCs, an embryonic tumor cell line, testes, ovaries, spleen, and placenta (Figure 1B). As a result, the expression pattern of ECAT11 seems to be similar between mice and humans.

### Generation of ECAT11-EGFP knock-in mice and ESCs

**Figure 2:**
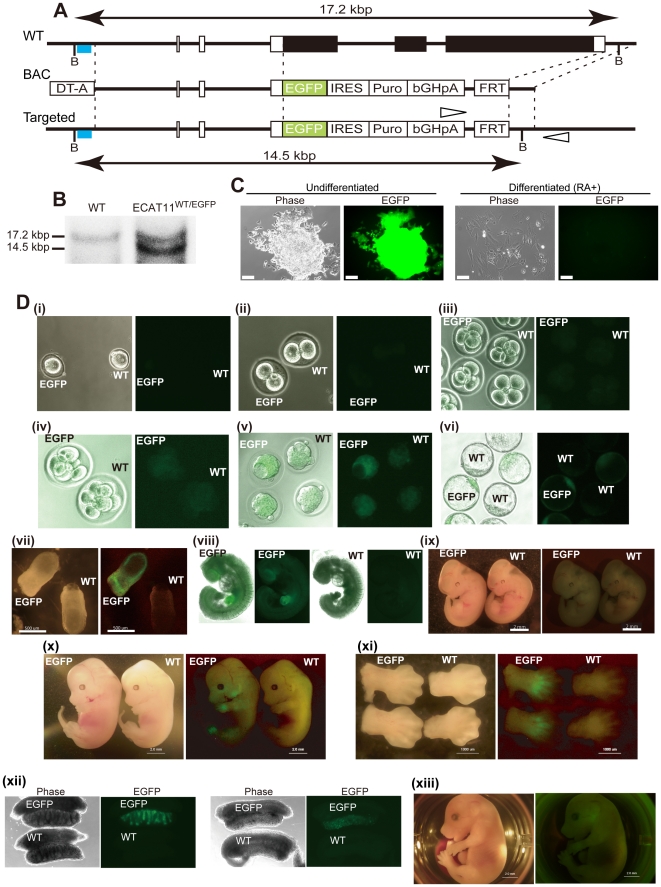
Generation of the ECAT11-EGFP knock-in reporter and mice. (A) Schematic representations of the structures of the mouse ECAT11 gene (WT), targeting BAC (BAC), and targeted loci (Targeted). The positions of the probes (blue lines), and recognition sites of BspHI (B) are shown. The black box indicates the ECAT11 coding region. Open arrowheads indicate the 3′ screening primer pair that amplified only the recombinant allele. DT-A: diphtheria toxin A, IRES: internal ribosomal entry site, Puro: puromycin resistance gene, bGHpA: bovine growth hormone poly A sequence, FRT: flippase recognition target. (B) The results of the Southern blot analysis of ECAT11^WT/EGFP^ ESCs. (C)The morphology of the ECAT11^EGFP/EGFP^ ESCs grown on gelatin-coated dishes. The phase and EGFP fluorescence images of undifferentiated (left panels) and differentiated (right panels) cells are shown. Cells were cultured with RA for eight days before taking the photographs. Bars; 100 µm. (D) ECAT11-EGFP expression in mouse embryos. Bright field and EGFP fluorescence images of fertilized eggs (i), 2 cells (ii), 4 cells (iii), 8 cells (iv), morulae (v), blastocysts (vi), E7.5 gastrulae (vii), E9.5 embryos (viii), E10.5 embryos (ix), E13.5 embryos (x), limbs of E13.5 embryos (xi), reproductive glands of E13.5 embryos; left, testis; right, ovary (xii), and the E15.5 embryo of an ECAT^EGFP/EGFP^ mouse (xiii) are shown. WT: wild type. EGFP: ECAT11^EGFP/EGFP^.

To elucidate the effect of ECAT11disruption and pursue its expression, we generated an ECAT11-EGFP knock-in construct on a bacterial artificial chromosome (BAC) vector. We first replaced the protein cording region of ECAT11 with anEGFP-IRES-Puro cassetteby enzyme-mediated recombination (Figure 2A). The manipulated BAC was then introduced into ESCs by electroporation. After drug selection, we obtained 750 drug resistant colonies. We first screened for the recombination by genomic PCR and then confirmed the recombination by Southern hybridization, in which the wild-type and targeted locus gave rise to bands of 17.2 kbp and 14.5 kbp, respectively (Figure 2B). We found that one out of the 750 clones had the correct homologous recombination. The ECAT11^WT/EGFP^ ESCs were positive for GFP fluorescence, but became negative when differentiation was induced by retinoic acid treatment (Figure 2C), thus suggesting that our reporter recapitulated the endogenous expression. By introducing the ESC clone into blastocysts, we established chimeric, and subsequently ECAT11 knock-in, heterozygous mice.

### ECAT11-EGFP localization in mouse embryos

To study the expression of ECAT11 during mouse development, we observed the developmental process from egg to embryonic day (E) 15.5 embryos using ECAT11-EGFP expression (Figure 2D). While EGFP fluorescence was not observed until themorula stage, an obvious expression was noted in the whole blastocyst from the blastula stage (Figure 2D (i)–(vi)). The expression was conspicuous in both embryonic and extraembryonic tissue onE7.5 (Figure 2D (vii)). GFP fluorescence gradually decreased until E9.5, and had completely disappeared by E10.5 (Figure 2D (viii), (ix)). The signal of ECAT11-EGFP appeared again at E13.5 inthe interdigitregions and lower jaw (Figure 2D (x), (xi)). ECAT11-EGFP expression was also observed in the testes and ovaries (Figure 2D (xii)). The fluorescence in the lower jaw and inter-digit regions was detectable until E15.5 (Figure 2D (xiii)).

### The effects of ECAT11 disruption in the entire mouse and ESCs

To elucidate the effects of ECAT11 disruption, we compared the ECAT11^EGFP/EGFP^ and WT mice. The interbred ECAT11 ^WT/EGFP^ mice yield F1 pups, including ECAT11 null mice, according to the Mendelian rule (Wild-type∶ECAT11^WT/EGFP^∶ECAT11^EGFP/EGFP^ = 43∶74∶46). ECAT11-null mutant mice were normal in gross appearance and by the X-ray analyses(Figure S1), and were found to be fertile. Therefore, ECAT11^EGFP/EGFP^ is dispensable in mouse development and fertilization.

**Figure 3:**
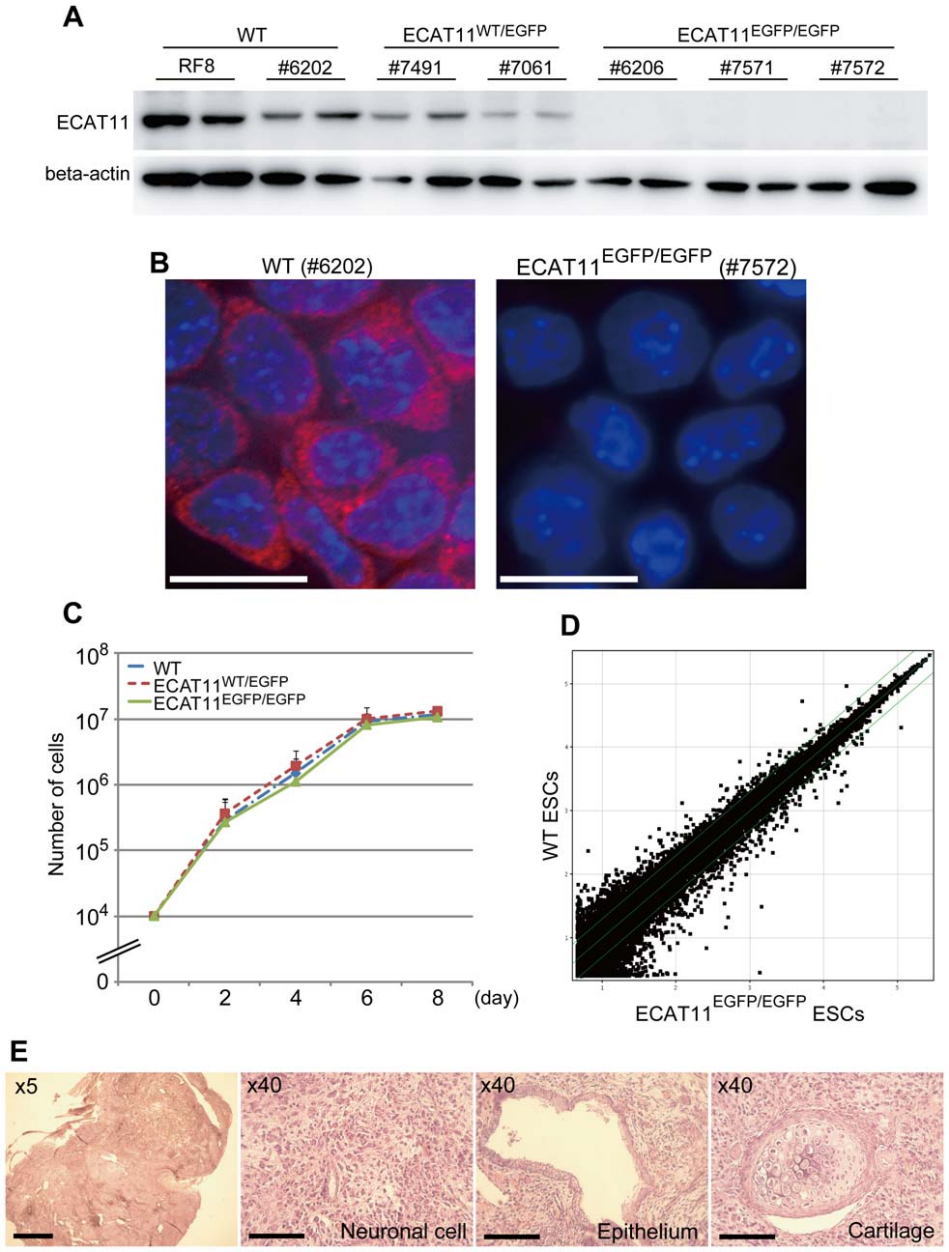
ECAT11 is dispensable for mouse ESCs. (A)A Western blot analysis of the ECAT11 expression in ESC lines. The data are shown in duplicate. (B) Immunocytochemistry of ECAT11. Red: ECAT11, Blue: DAPI. Bars; 20 µm. (C)The proliferation of ECAT11 knock-in ESCs. Ten thousand cells were plated, and counted on days2, 4, 6 and 8. We used 2 WT, 2 ECAT11^WT/EGFP^, and 3 ECAT11^EGFP/EGFP^ ESC lines for this analysis. The data are shown as the averagesand standard deviations. (D) Scatter plots showing a comparison of the global gene expression determined by the microarray analyses between the WT and ECAT11^EGFP/EGFP^ ESCs. The data from 2 WT ESC lines and 3 ECAT11^EGFP/EGFP^ ESC lines cultured on gelatin coated dishes were averaged and used for this analysis. (E)Hematoxylin and eosin staining of teratomas generated from ECAT11^EGFP/EGFP^ ESCs. The gross image (upper left), neural tissue, gut-like epithelia, and cartilage are shown. The scale bars are 200 µm in the gross image and 100 µm in the other images.

To investigate the functions ofECAT11 in ESCs, we obtained 25 blastocysts from intercrosses of ECAT11^WT/EGFP^ heterozygous mice and established 14 ESC lines. Among them, two lines were wild type, nine were ECAT11^WT/EGFP^, and 3 were ECAT11^EGFP/EGFP^. We usedonewildtype line (#6202), two ECAT11^WT/EGFP^ lines (#7491 and #7061), and three ECAT11^EGFP/EGFP^ lines (#6206, #7571 and #7572), as well as the RF8 parental ESC line, in the subsequent analyses. A Western blot analysis confirmed the absence of ECAT11 expression in the ECAT11^EGFP/EGFP^ ESC lines (Figure 3A). Immunostaining also confirmed the absence of the ECAT11 protein in ECAT11^EGFP/EGFP^ ESC lines (Figure 3B). In wild-type ES cells, immunofluorescent microscopy using an anti-ECAT11 antibody detected ECAT11 in the cytoplasmin a spotty pattern, which is similar to that of the L1ORF1 protein (Figure 3B) [22]. No such signal was detected in the ECAT11^EGFP/EGFP^ ESC lines (Figure 3B).

ECAT11^EGFP/EGFP^ ESCs showed normal morphology and proliferation (Figure 3C). They also showed similar global gene expression profiles asthe wild type ESCs as determined by a microarray analysis (Figure 3D). ECAT11^EGFP/EGFP^ ESCs did not exhibit significant change in comparison with wild-type ESCs (>2-fold, p<0.05). When subcutaneously transplanted into immunodeficient mice, ECAT11^EGFP/EGFP^ ESCs formed teratomas, which consisted of various tissues of all three germ layers, such as neuronal cells, epithelium, and cartilage (Figure 3E). Therefore, ECAT11 is also dispensable for theself-renewal of pluripotent ESCs.

### Induction of ECAT11 expression in mouse embryonic fibroblasts

**Figure 4:**
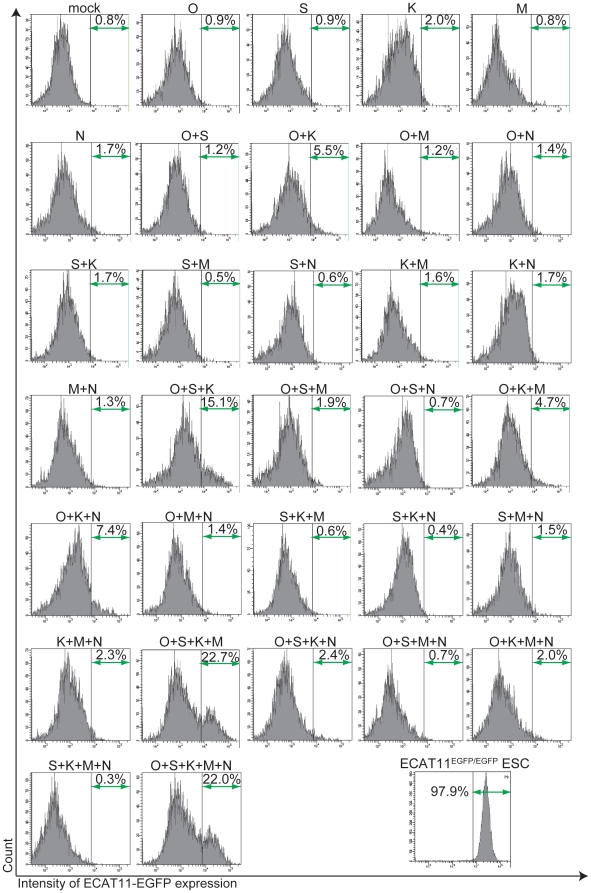
Activation of the ECAT11 promoter by forced expression of Oct4, Sox2 and Klf4. The expression of the ECAT11-EGFP fluorescent marker was induced by various combinations of transcription factors. EGFP fluorescence was analyzed by flowcytometry two days after retroviral transduction. O: Oct4, S: Sox2, K: Klf4, M: c-Myc, N: Nanog.

We examined whether ECAT11 can be activated by pluripotency-associated transcription factors (Oct4 (O), Sox2 (S), Klf4 (K), c-Myc (M) and Nanog (N)) in mouse embryonic fibroblasts (MEFs). We introduced various combinations of the five transcription factors into MEFs established from ECAT^EGFP/EGFP^ embryos (Figure 4). Two days after the transduction of 31 possible combinations, we examined the expression of ECAT11-EGFP by a flow cytometer. No single factor was able to induce EGFP fluorescence. However, we observed arapid and robust (>10%) activation by three combinations, OSK, OSKM, and OSKMN (Figure 4). Modest activation (∼5%) was obtained by OK, OKN, and OKM. These data indicated that ECAT11 is rapidly activated by Oct3/4 and Klf4, together with Sox2.

### ECAT11 is dispensable for iPSC generation

**Figure 5:**
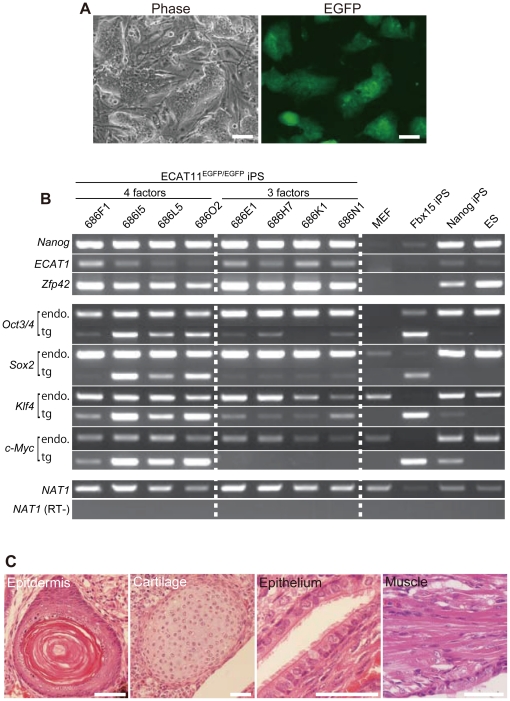
Generation of iPSCs from ECAT11^EGFP/EGFP^ MEFs. (A) Morphology of an ECAT11^EGFP/EGFP^ iPSC colony, which was picked on day 23 after induction of four factors (Oct4, Sox2, Klf4 and c-Myc) and cultured on feeder cells for three passages. Scale bars: 100 µm. (B) The expression levels of three pluripotency markers (Nanog, ECAT1 and Zfp42), and four transcription factors (Oct4, Sox2, Klf4 and c-Myc). Total RNA was collected from four clones of ECAT11^EGFP/EGFP^ iPSCs(686F1, 686I5, 686L5 and 686O2) established using four factors (Oct4, Sox2, Klf4 and c-Myc), and four clones (686E1, 686H7, 686K1 and 686N1)established usingthree factors (Oct4, Sox2 and Klf4). The iPSCs selected with Fbx15 orthe Nanog reporter (20D17), MEFs, and ES cells were also usedas controls. For reprogramming factor detection, RT–PCR analyses were performed with primers that amplified endogenous transcripts only (endo) and transgene transcripts only (tg) to detect the viral vector silencing. (C) Hematoxylin and eosin staining of teratomas generated from ECAT11^EGFP/EGFP^ iPSCs. Scale bars: 50 µm.

The rapid activation of ECAT11 in MEFs by OSK(M) prompted us study whether iPSCs can be generated without ECAT11. We introduced the four reprogramming factors (OSKM) by retroviruses into ECAT11^EGFP/EGFP^ MEFs. Five days after transduction, cells were re-seeded onto SNL feeder cells and selected with puromycin. Approximately 20 days after transduction, we observed many puromycin-resistant colonies. These cells were expandable, and showed a morphology and proliferation similar to ESCs (Figure 5A). These cells expressed pluripotency-associated genes, such as Nanog, at comparable levels to those in ES cells (Figure 5B). They also formed teratomas containing various tissues representing all three germ layers (Figure 5C). These data demonstrated that ECAT11 is dispensable for mouse iPSC generation.

## Discussion

In this study, we generated ECAT11-EGFP knock-in/-out mice and studied the expression and functions of ECAT11. We confirmed that ECAT11 is expressed in early mouse embryos and undifferentiated ES cells. We also found that ECAT11 is rapidly activated during iPSC generation. Despite this specific expression, ECAT11-deficient ES cells were normally self-renewed and remained pluripotent. We were able to generate iPSCs from ECAT11-null fibroblasts. These data demonstrated that ECAT11 is dispensable for the induction and maintenance of pluripotency, despite its specific expression.

It has been reported that a lot of truncated sequences derived from L1 are dispersed in the mouse genome [25], indicating that these fragments might work as complementary factors for ECAT11. Indeed, some dispersed L1 sequences are still active in several types of somatic cells [26],[27] and germ cells at various developmental stages [28]. L1 expression was also observed in the blastocyst, from which ESCs are derived [29]. The L1ORF1 protein binds to RNA in a sequence non-specific manner [30]. In addition, other putative proteins containing Transposase_22 are interspersed in the mouse genome (EMBL-EBI: IPR004244 Transposase_22: http://www.ebi.ac.uk/interpro/IEntry?ac=IPR004244). Therefore, these related proteins might compensate for a loss of function of the ECAT11 protein.

The expression of ECAT11-EGFP in MEFs was effectively promoted by the forced expression of three factors, Oct4, Sox2 and Klf4. This induction was further enhanced by c-Myc. Previous reports of studies using chromatin-immunoprecipitation assays showed that the promoter region of ECAT11 is occupied by Klf4 and c-Myc in mouse ESCs [31],[32]. Oct4 and Sox2 have been shown to regulate the transcriptional activity of target genes through the interaction with their recognition sequences, octamer- and SRY-binding sites, respectively. The database analysis of transcription factor binding sites using TFSEARCH (http://www.cbrc.jp/research/db/TFSEARCH.html) identified multiple octamer- and SRY-binding sites in the 5′- flanking region of the mouse ECAT11 gene. However, genome wide mapping of Sox2 and Oct4 binding sites by ChIP-seq could not detect their interaction in these regions in ESCs [32]. It therefore remains to be determined whether Oct3/4 and Sox2 directly activate the transcription of ECAT11.

In conclusion, we have herein demonstrated that ECAT11 disruption does not affect the function of ESCs, mouse development or fertility. By using a reporter mouse, we found that the ECAT11 promoter is rapidly activated by ectopic expression of Oct4, Sox2 and Klf4. Nevertheless, iPSCs can be generated from ECAT11-null fibroblasts. Therefore, ECAT11/L1ltd1 is considered to be dispensable for the induction and maintenanance of plutipotency, despite its specific expression.

## Materials and Methods

### Cell culture, induction of transcription factors and reprogramming

ESCs (RF8mouse ES cell line [33] and all other embryonic stem cell lines established in this research)were maintainedin DMEM supplemented with 20% FBS(Invitrogen), 0.1 mM non-essential aminoacids(Invitrogen), 2 mM L-glutamine(Invitrogen), 50 U/ml penicillin-streptomycin(Invitrogen), 0.11 mM 2-mercaptoethanol(Invitrogen)and LIF on feeder layers of mitomycin C-treated SNL cells [34] or gelatin coated dishes. As a source of leukemia inhibitory factor (LIF), we used conditioned medium (1∶1000 dilution) from Plat-E cell cultures that had been transduced with a LIF expression vector. ESCs were passaged every 2 days. Plat-E packaging cells, which were also used to produce retroviruses, were maintained in DMEM containing 10% FBS, 50 units/50 mg/ml penicillin/streptomycin, 1 µg/ml puromycin (Sigma), and 10 µg/ml of blasticidin S (Funakoshi). H9 human embryonic stem cells (WiCell) were maintained in Primate ES medium(ReproCELL, Japan) supplemented with 4 ng/ml recombinant humanbasic fibroblast growth factor (bFGF, WAKO, Japan). Retroviral transductions and induction of nuclear reprogramming were performed as described previously [35].

### Mice

All mice used in this study were bred and sacrificed appropriately following code of ethics of animal research committee in Kyoto University.The animal care and experimental procedures of this subject were approved by the Animal Research Committee, Kyoto University and carried out according to the Regulation on Animal Experimentation at Kyoto University (approval ID : I-6-5).

### Construction of targeting vectors for targeted disruption of ECAT11 in mouse ESCs

We purchasedthe bacterial artificial chromosome (BAC) clone RP24-326M13 containing ECAT11 from the BACPAC resources center. By using the RED/ET recombination technique (Gene Bridges), we replaced from the translation initiation site to exon 5 that contained the ECAT11 coding region with an EGFP-IRES-Puro cassette and inserted a diphtheria toxin A cassette 8.7 kbp upstream of the 5′ arm. After linearization with BspTI, the modified targeting vectors were introduced into RF8 ESCs by electroporation. Genomic DNA isolated from puromicin-resistant colonies was screened for homologous recombination by PCR, and correct targeting was confirmed by a Southern blot analysis of BspHI-digested DNA with a specific probe. The Southern blot analysis was performed following the manufacturer's protocols. For the sequences of the primers and probes, see Table 1.

**Table 1:** PCR primers and probes list.

Subject	gene or target name	sense primer	anti-sense primer
RT-PCR	ECAT11 (mouse)	TCTCAGACCTTCACAGCCTCCGTC	CCCCTCCTGCTTCTTGAGAGTTCC
	ECAT11 (human)	CATTTTCAGATCTGCAAAGCCTTAG	TTCATCTCCTCTGGCTTAGCAAC
	NAT1 (mouse and human)	ATTCTTCGTTGTCAAGCCGCCAAAGTGGAG	AGTTGTTTGCTGCGGAGTTGTCATCTCGTC
	Nanog	AGGGTCTGCTACTGAGATGCT	CAACACCTGGTTTTTCTGCCACCG
	ECAT1	TGT GGG GCC CTG AAA GGC GAG CTG AGA T	ATGGGCCGCCATACGACGACGCTCAACT
	Zfp42	ACGAGTGGCAGTTTCTTCTTGGGA	TATGACTCACTTCCAGGGGGCACT
	Oct4 endo	TCTTTCCACCAGGCCCCCGGCTC	TGCGGGCGGACATGGGGAGATCC
	Oct4 Tg	TTGGGCTAGAGAAGGATGTGGTTC	TTATCGTCGACCACTGTGCTGCTG
	Sox2 endo	TAGAGCTAGACTCCGGGCGATGA	TTGCCTTAAACAAGACCACGAAA
	Sox2 Tg	GGTTACCTCTTCCTCCCACTCCAG	TTATCGTCGACCACTGTGCTGCTG
	Klf4 endo	CCAACTTGAACATGCCCGGACTT	TCTGCTTAAAGGCATACTTGGGA
	Klf4 Tg	GCGAACTCACACAGGCGAGAAACC	TTATCGTCGACCACTGTGCTGCTG
	c-Myc endo	TGACCTAACTCGAGGAGGAGCTGGAATC	TTATGCACCAGAGTTTCGAAGCTGTTCG
	c-Myc Tg	CAGAGGAGGAACGAGCTGAAGCGC	TTATCGTCGACCACTGTGCTGCTG
BAC modification	ECAT11 region	TTCTGACGCTCTCGCCAGGAAAAGGTGCGCTTTGTGTCAGTGTATCCACCTCGCCACCATGGTGAGCAAG	AGGTTCTCTGGAAAATAAGCAGGGACATTAAGTAAACCAATGACAAATGCAGTTTATGGCGGGCGTCCTG
	DTA for ECAT11 upstream region	GATGTGTGCCACCGTGCCCAGCAACCCACTCTTCTATTAATTAATTAACTAGCGCGCAATTAACCCTCAC	GGTGCACACACATATGTGCAGGTAAATACATATTTAATTAATTAACTAGTGCGCGCGTAATACGACTCAC
Recombinant allele screening	ECAT11 3' region	TCGCATTGTCTGAGTAGGTGT	GAATAGCCAAGCAACTTCTACTGT
Genotyping	ECAT11	TCCAGGAGCACATCACATTAGTGACCC	ATTGCATCTCCTTCTTGCTGCCCTATC
			CGTCGCCGTCCAGCTCGACCAG
Southern hybridization	ECAT11 5' region	CTGAACCCCAGTTCTCCCAACC	CCTTAGGTCAGGCATATGCGAATA

### Generation of ECAT11-deficient mice and ESCs

The ECAT11 disrupted (ECAT11^WT/EGFP^) heterozygous ESCs were microinjected into C57BL/6 blastocysts and implanted into pseudopregnantJcl:ICR females to obtain chimeric mice. The chimeric founders were mated with each other or C57BL/6 mice to generate heterozygous ECAT11^WT/EGFP^ mice, which were then intercrossed to produce homozygous ECAT11^EGFP/EGFP^ mice. All of the phenotype analyses were performed with littermates on a mixed 129/Sv and C57BL/6 background.

To establish ECAT11^EGFP/EGFP^ ESCs, ECAT11^WT/EGFP^ or ECAT11^EGFP/EGFP^ female mice were injected with 10 µg of tamoxifen (Sigma) and 1 mg of depo-provera (Sigma) subcutaneously on the third day after mating. Four days later, pregnant mice were sacrificed, and embryos in diapause were flushed out of the uterus by PBS supplemented with 10% FBS. These blastocysts were cultured on SNL feeder cells in four-well plates in DMEM supplemented with 10% FBS, 0.1 mM non-essential aminoacids, 2 mM L-glutamine, 50 U/ml penicillin-streptomycin, and 0.11mM 2-mercaptoethanol. After 6 days, the central mass of each explant was harvested, rinsed in PBS, and placed in a drop of trypsin for 5 minutes. The cell mass was collected with a finely drawn-out Pasteur pipette preloaded with medium, ensuring minimal carryover of the trypsin. The cells were gently transferred into a fresh well with medium containing 20% FBS. The resulting primary ESC colonies were individually passaged into the wells of four-well plates containing SNL feeder cell layers. Thereafter, cells were expanded by trypsinization of the entire culture. Genotypes of established clones were confirmed by genotyping PCR (For primers, see Table 1).

### RT-PCR and real-time PCR

Total RNA from mice was purified with the TRIzol reagent (Invitrogen) and treated with a Turbo DNA-free kit (Ambion) to remove genomic DNA contamination. Total RNA of dermal fibroblasts and various tissues from adult humans were purchased from STRATAGENE® and CELL APPLICATIONS, Inc. One microgram of total RNA was used for the reverse transcription reaction with ReverTraAce-A (Toyobo, Japan) and the dT20 primer, according to the manufacturer's instructions. PCR was performed with Ex Taq HS (Takara, Japan).

### Generation of anti-ECAT11 polyclonal antibodies

The coding sequence of aa275 to 360 of *ECAT11* was amplified by PCR (For primers, see Table 1). The PCR product was subcloned into pENTR/D-TOPO (Invitrogen), and was transferred into pDEST17 (Invitrogen) by LR recombination. After introduction of the resulting expression vector, pDEST17- mECAT11-275360, into BL21-AI *E. coli* (Invitrogen), recombinant protein production was induced according to the manufacturer's protocol. Histidine-tagged ECAT11 was purified using Ni-nitrilotriacetic acid agarose (Qiagen) under denaturing conditions in the presence of 8 M urea. After dialysis against 6 M urea, the recombinant proteins were consigned to SCRUM Inc. to generate anti-ECAT11 rabbit polyclonal antibodies.

### Western blot analysis

The cells in a semiconfluent state were lysed with M-PER (Thermo Scientific) supplemented with protease inhibitor cocktail (Roche). Ten microliters of the cell lysate were separated by electrophoresis on 8% SDS-polyacrylamide gels, then were transferred to a polyvinylidinedifluoride membrane (Millipore), and probed with the primary antibody against ECAT11 (1∶1000) or β-actin (1∶5000, A5441, Sigma). Signals were detected with anti-mouse IgG-HRP (1∶3000, #7076, Cell Signaling) or anti-rabbit IgG-HRP (1∶2000, #7074, Cell Signaling).

### Immunocytochemistry

ESCs were fixed with PBS containing 4% paraformaldehyde for 20 min at room temperature. After washing with PBS, the cells were treated with PBS containing 5% normal goat serum (Millipore), 1% bovine serum albumin (BSA, Nacalaitesque), and 0.2% Triton X-100 for 45 min at room temperature. After 3 washed with PBS, 8 µl of primary antibody (anti-ECAT11 rabbit whole serum) was diluted in 800 µl of PBS containing 1% BSA and added into blocked cells. The secondary antibody used was cyanine 3 (Cy3)-conjugated goat anti-Rabbit IgM (1∶500, Millipore).

### Teratoma formation

The cells were harvested by 0.25% trypsin/1 mM EDTA treatment, collected into tubes, and centrifuged, and the pellets were suspended in 10% FBS/DMEM. One million of the cells were injected subcutaneously into the dorsal flank of a nude mouse (CREA, Japan). Eight weeks after the injection, tumors were dissected, weighed, and fixed with PBS containing 4% paraformaldehyde. Paraffin-embedded tissues were sliced and stained with hematoxylin and eosin.

### Cell proliferation assay

Ten thousand cells were plated in duplicate onto 60 mm gelatin coated dishes. Before counting cells, these cultured dishes were washed with 1 ml PBS and cells were dispersed with 500 µl of 0.25% trypsin. Thereafter, 2 ml of medium were added, and 100 µl of the suspension was used for counting. The number of cells was counted on days 2, 4, 6, and 8 using a Z1 Coulter Particle Counter (Beckman coulter). This procedure was repeated three times.

### Microarray analysis

Total RNA from wild-type and ECAT11^EGFP/EGFP^ ESCs were labeled with Cy3, and were hybridized to oligonucleotide microarrays (Agilent) according to the manufacturer's protocol. Hybridization was repeated with ESCs representing two independent wild-type clonesand three ECAT11^EGFP/EGFP^ clones. The arrays were scanned with a G2565BA Microarray Scanner System (Agilent). The data were analyzed using the GeneSpringGX ver. 11.5 software program (Agilent). Each chip was normalized to the 75th percentile of the measurements. The flag settings were set as described below. Absent;Spot feature is not uniform, saturated or population outlier, Marginal; spot feature is not positive or not above background:, Present;spot feature is other than those above. The genes having present flag in at least one out of the five samples wereused for the analyses.We compared wild-type and ECAT11^EGFP/EGFP^ ESCs and analyzed the result based on unpaired t-statistics with Benjamini and Hochberg false discovery rate (>2-fold change, p<0.05). Microarray data are available in GEO (Gene Expression Omnibus, http://www.ncbi.nlm.nih.gov/projects/geo/index.cgi) with the accession number GSE28145.

### MEF establishment

MEFs were derived from ECAT11^EGFP/EGFP^ embryos at E13.5. After removal of the head and gastrointestinal tract, the embryos were minced, and dissociated with trypsin. The cell suspensions were plated onto gelatin-coated dishes through 70 µm mesh. Five days after plating, the established MEFs were counted, and 5×10^6^ cells were stored using freezing medium. The MEFs were thawed and used for induction assays after one passage.

### FACS analysis

Cells were harvested by incubation in 0.25% trypsin/1 mM EDTA for 5 min at 37°C, and single-cell suspensions were obtained by repetitive pipetting and transfer through a 70 µm cell strainer. After washing with PBS supplemented with 3% FBS, cells were resuspended in PBS containing 1/1000 volumes of DAPI and analyzed by a FACSAria II instrument (BD Biosciences). Dead cells were excluded by staining with DAPI. The data were analyzed with the Diva 6.1 software program (BD Biosciences).

## Supporting Information

Figure S1 **X-ray analysis of ECAT11^EGFP/EGFP^ mice.** X-ray evaluation of ECAT11^EGFP/EGFP^ or WT mice (2-week-old).
(TIF)
