# Supplementary figures and images for: ECAT11/L1td1 Is Enriched in ESCs and Rapidly Activated During iPSCGeneration, but It Is Dispensable for the Maintenance and Induction of Pluripotency
Source: PLoS One. 2011 May 26;6(5):e20461. doi: 10.1371/journal.pone.0020461 (PMC3102727; doi:10.1371/journal.pone.0020461)

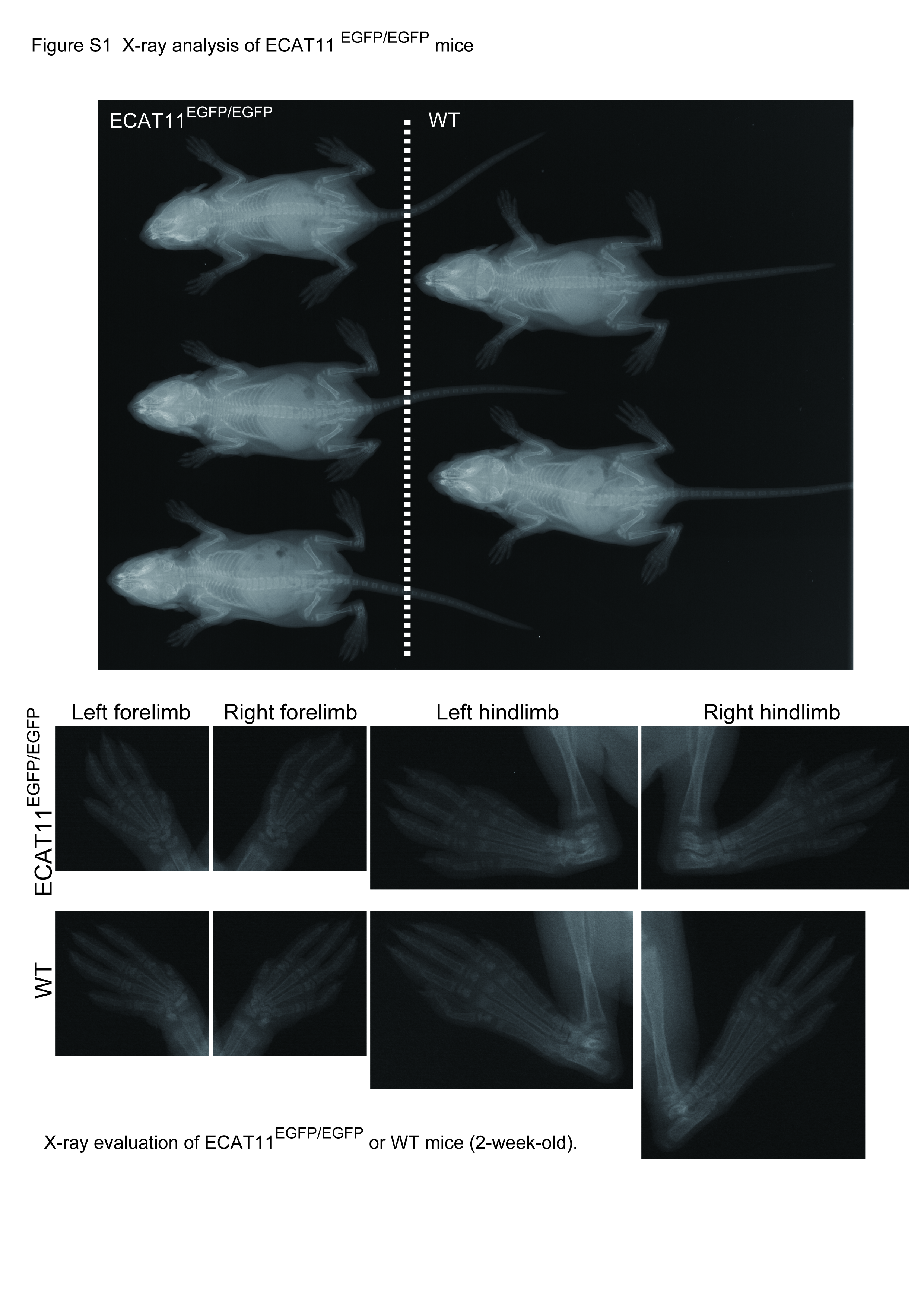

Supplement: Figure S1 — X-ray analysis of ECAT11EGFP/EGFP mice. X-ray evaluation of ECAT11EGFP/EGFP or WT mice (2-week-old). (TIF) [file pone.0020461.s001.tif]
